# Kugel’s Artery: Silent Bystander or Savior?

**DOI:** 10.7759/cureus.17039

**Published:** 2021-08-09

**Authors:** Joshua T Narh, Khushal V Choudhary, Adeniyi Okunade, Kriti Lnu, Anoop Puskoor, Yuvraj Chowdhury, Erdal Cavusoglu

**Affiliations:** 1 Internal Medicine, One Brooklyn Health - Brookdale University Hospital and Medical Center, Brooklyn, USA; 2 Internal Medicine, Roger Williams Medical Center, Providence, USA; 3 Internal Medicine, University of Pittsburgh Medical Center Pinnacle, Harrisburg, USA; 4 Internal Medicine, State University of New York (SUNY) Downstate Medical Center, Brooklyn, USA; 5 Cardiovascular Medicine, State University of New York (SUNY) Downstate Medical Center, Brooklyn, USA

**Keywords:** kugel’s artery, coronary artery disease, acute coronary syndrome, percutaneous coronary intervention, chronic total occlusion

## Abstract

Kugel’s artery is defined as a rare anatomical variant of the coronary artery vascular bed consisting of an anastomotic connection between branches of the right coronary artery (RCA) and/or left circumflex artery (LCX). Kugel’s artery has been reported to have an incidence of 6% in the general population. The presence of this anastomotic communication may play a pathophysiological role in a patient with a right dominant coronary circulation and an underlying coronary artery disease (CAD) affecting the right coronary system. Understanding the existence and significance of Kugel’s artery and the anastomotic network cannot be overemphasized. The presence of an anomalous vascular connection bypassing an area of epicardial vessel occlusion may be a lifesaving pathophysiological finding that maintains myocardial perfusion and viability. Herein, we present a case with multivessel occlusion myocardial infarction found to have anomalous vascular anastomosis between the proximal RCA and distal segment of the same artery.

## Introduction

Kugel’s artery is defined as a rare anatomical variant of the coronary artery vascular bed consisting of an anastomotic connection between branches of the right coronary artery (RCA) and/or left circumflex artery (LCX) [1]. Due to its relevance to coronary artery circulation and morphological characteristics, it was originally termed “arteria anastomotica auricularis magna’ [2,3]. Kugel’s artery has been reported to have an incidence of 6% in the general population [4]. Anatomically, this intravascular conduit may arise from the proximal RCA and terminate in the distal segment of the same artery; from the proximal LCX to the RCA; LCX to the distal segment of the same artery. A majority of cases are detected by coronary angiogram [4,5]. The presence of this anastomotic communication may play a pathophysiological role in a patient with a right dominant coronary circulation and an underlying coronary artery disease (CAD) affecting the right coronary system. There exist only a few reported cases of Kugel’s artery with the pathophysiological relationship in CAD remaining unclear. Herein, we present a case with multivessel occlusion myocardial infarction found to have anomalous vascular anastomosis between the proximal RCA and distal segment of the same artery. We also aim to review the literature and attempt to explain the physiological relevance of Kugel’s artery.

## Case presentation

**Figure 1:**
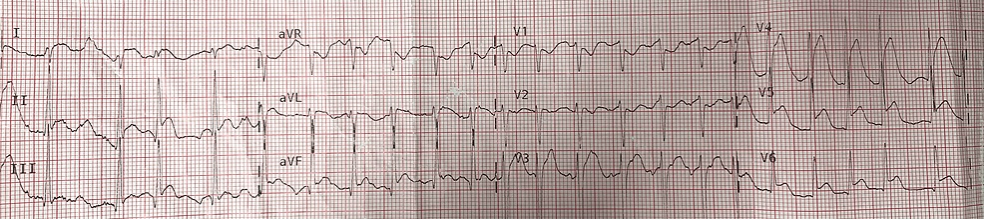
Post resuscitation 12-lead EKG done by emergency medical services (EMS) in the field.

**Figure 2:**
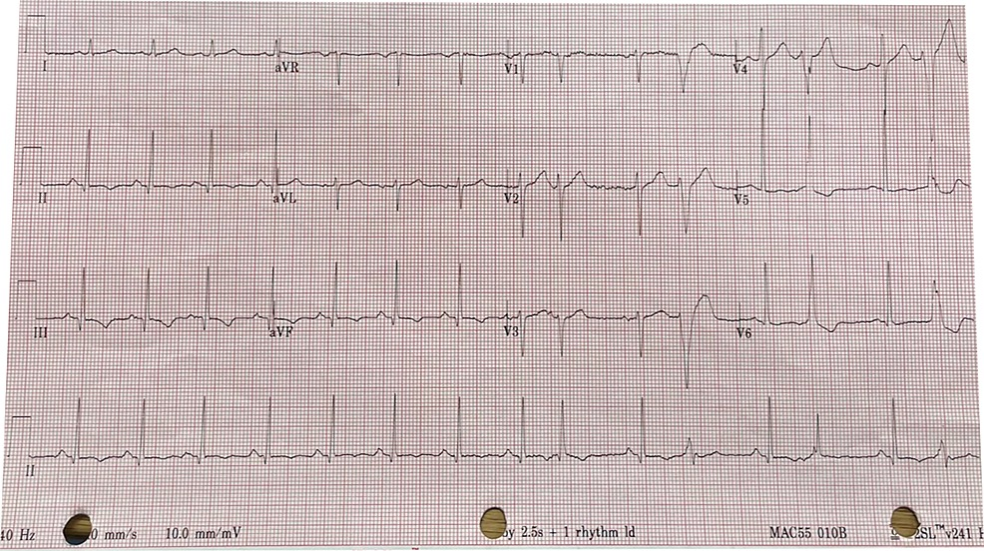
12-lead EKG done on arrival in the emergency room.

A 67-year-old African American male with no significant past medical history presented to the emergency room for an out-of-hospital witnessed cardiac arrest. The patient had completed a treadmill exercise lasting for 30 minutes without limiting symptoms and was recovering in a sitting position when he suddenly became unresponsive and pulseless. His spouse called emergency medical services (EMS) and initiated cardiopulmonary resuscitation (CPR). Upon EMS arrival, the patient was found to be in ventricular fibrillation requiring one episode of 200 J of unsynchronized cardioversion with successful return of spontaneous circulation (ROSC). Estimated downtime without CPR was approximately two minutes. Post-ROSC 12-lead electrocardiogram (EKG) demonstrated sinus rhythm, 2-mm ST-segment elevation in lead I, II, III, AVF, V3, V4, V5, and V6 (Figure 1). He was intubated in the field and brought forthwith to the emergency room. EKG done on arrival in the emergency room showed sinus rhythm with premature atrial complexes, without notable ST-segment elevation (Figure 2). Severe acute respiratory syndrome‐coronavirus 2 (SARS‐CoV‐2) polymerase chain reaction (PCR) nasopharyngeal swab revealed negative reactivity. The rest of the laboratory findings are stated in Table 1.

**Table 1:** Laboratory findings on arrival in the emergency room. ALP: Alkaline Phosphatase; ALT: Alanine aminotransferase; AST: Aspartate aminotransferase; BNP: Brain Natriuretic Peptide; BUN: Blood urea nitrogen; Cl: Chloride; CPK: Creatine kinase; Cr: Creatinine; Hgb: Hemoglobin; K: Potassium; LDL-C: Low density lipoprotein cholesterol; Na: Sodium; WCC: White cell count.

Hematology		Biochemistry			
White blood cell	7.8	Na^+^ mmol/L	139	Troponin ng/ml	2.96
Neutrophils %	43.5	K^+^ mmol/L	3.4	BNP pg/ml	246
Lymphocyte %	47.2	Cl^-^ mmol/L	104	AST U/L	283
Hgb g/dl	13.7	BUN mg/dl	14	ALT U/L	317
Platelet x 10^9/L	209	Cr mg/dl	1.2	ALP U/L	66
		Phosphorus mg/dl	3.5	CPK U/L	166
		LDL-C	75		

**Figure 3:**
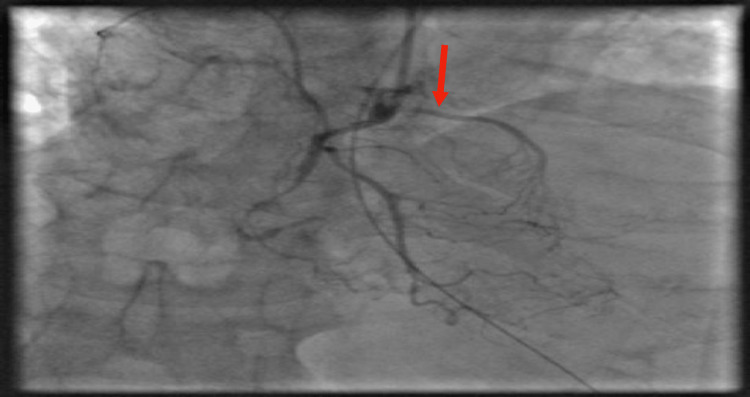
Coronary angiogram of the right coronary system in the right anterior oblique view showing CTO of mid-RCA and Anomalous (Kugel’s artery) vessel connection between proximal RCA and distal RCA (red arrow). CTO: Complete total occlusion; RCA: Right coronary artery.

**Figure 4:**
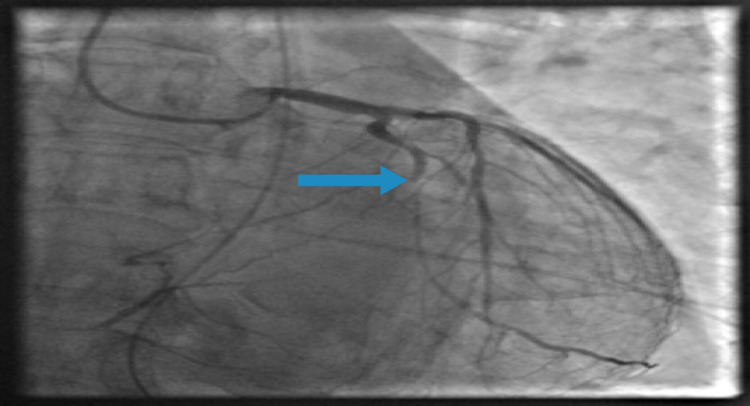
Coronary angiogram of the left coronary system in the right anterior oblique view showing 100% stenosis of the second obtuse marginal branch of the left circumflex artery (OM-2) (blue arrow).

**Figure 5:**
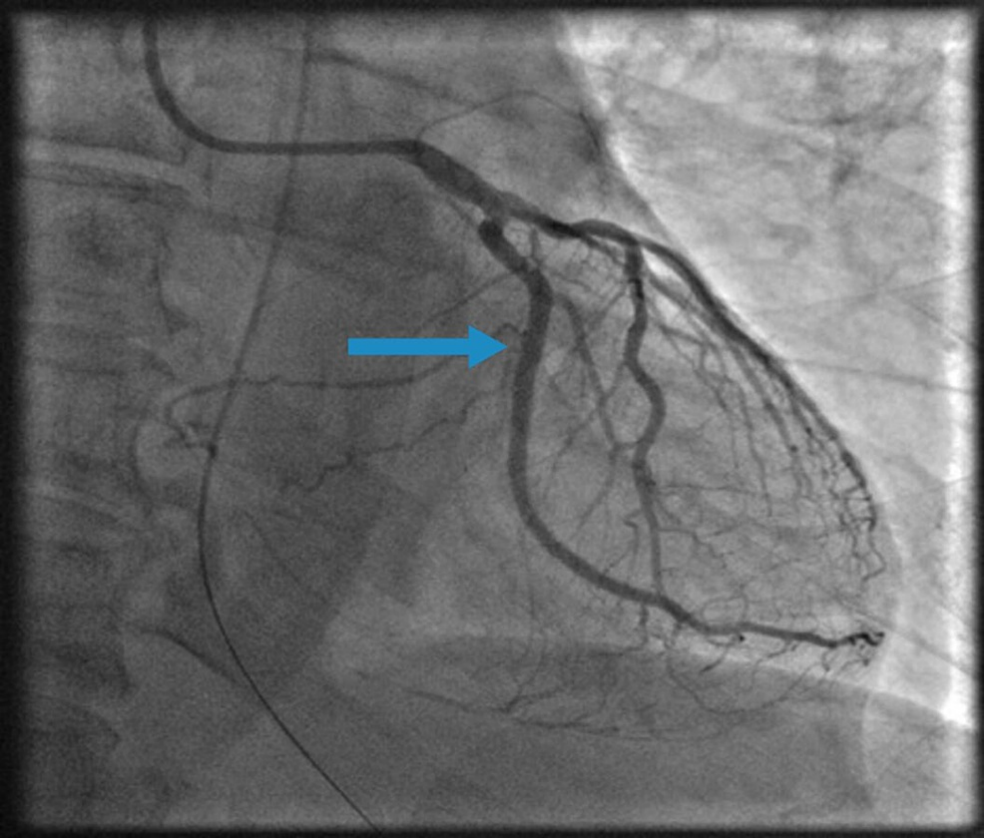
Coronary angiogram of the left coronary system in the right anterior oblique view. Following intervention there was an excellent angiographic appearance with a 0% residual stenosis of the OM-2. OM-2: Second obtuse marginal branch.

ST-elevation myocardial infarction (STEMI) alert was activated. The patient received aspirin, clopidogrel and was emergently sent to the cardiac catheterization laboratory for diagnostic angiogram and percutaneous coronary intervention (PCI). PCI revealed right dominant coronary circulation, patent left main (LM), 40% stenosis of the proximal obstruction of RCA, complete total occlusion (CTO) of middle RCA, and anomalous Kugel collateral vessel with roots from the proximal to the distal RCA (as seen in Figure 3), 30% stenosis of the proximal left anterior descending artery (pLAD), 30% to 40% stenosis of ostial LCX, 70% stenosis of middle LCX, subtotal stenosis of the second obtuse marginal branch of the LCX (OM-2) (Figure 4). Left ventricular (LV) ejection fraction was estimated at 40%, with notable postero-basal and diaphragmatic hypokinesis/akinesis by LV ventriculography. Hemodynamic measurements via right heart catheterization revealed pulmonary capillary wedge pressure (PCWP) of 38 mmHg, cardiac output (CO) of 5.5 L/min, cardiac index (CI) of 2.9 L/min2. A successful PCI, balloon angioplasty with deployment of three drug-eluting stents was performed on the 100% lesion in the large OM-2. Following intervention, there was an excellent angiographic appearance with a 0% residual stenosis (Figure 5).

Following the procedure, the patient was sent to the coronary care unit in a stable condition. Transthoracic echocardiogram (TTE) revealed LV ejection of 40%, mildly reduced systolic function, and inferior and inferoseptal hypokinesis, without significant valvular abnormalities.

## Discussion

Kugel’s artery is an atrial artery that connects directly or through the sinus node artery, the proximal part of the LCX or the RCA with the distal part of the vessel where it crossed the crux [6]. This artery is long and wide, and suitably fulfills the four initial descriptive terms “arteria anastomotica auricularis magna '' given by Kugel (Kugel, 1927). It can either arise from the LCX, penetrate the left atrial wall and proceed backward through the inferior border of the atrial septum to end at the crux. Alternatively, it can arise from the proximal RCA and terminate at the distal RCA or branches of the RCA. It can be formed by branches of the RCA and branches of the LCX that join near AV node artery.

In our case, the Kugel's artery was found to connect the proximal RCA to the branches of the distal RCA. The patient was found to have inferior anterolateral myocardial infarction on EKG and coronary angiogram revealed CTO of RCA and obtuse marginal. Despite total occlusion of the RCA one would expect to have a clinically significant myocardial infarction earlier in life however, because of the patent Kugel’s artery, the territory of RCA had viable blood supply and the presentation was most likely due to occlusion of the OM-2. This bears out the observation by previous studies that collateral circulation was not seen in angiography until the degree of arterial occlusion is greater than 90% [7,8]. A favorable long-term prognosis is associated with good collateralization in patients with angiographically-proven single or double vessel CAD [9]. Based on our patient’s EKG and coronary angiogram findings, his acute presentation was most likely due to myocardial infarction in the LCX territory. He denied previous cardiorespiratory symptoms which may indicate myocardial viability in the distribution of RCA maintained by the Kugel’s artery. Relevant clinical questions arise regarding the physiological effect of revascularizing, a non-culprit CAD lesion in the region of a Kugel’s artery. The possibility of a steal phenomenon may be likely between the Kugel’s artery and the affected epicardial artery. In our patient, there was no intervention to the total occlusion in the RCA.

## Conclusions

Understanding the existence and significance of Kugel’s artery and the anastomotic network cannot be overemphasized. The presence of an anomalous vascular connection bypassing an area of epicardial vessel occlusion may be a lifesaving pathophysiological finding that maintains myocardial perfusion and viability. Further studies will need to be pursued to have a better understanding into the pathophysiological importance of Kugel’s artery in acute coronary syndrome. Since the Kugel’s artery and the rest of the anastomotic network originates around the proximal end of the coronary trunks, it is crucial that interventional cardiologists and electrophysiologists who perform procedures in the region of the lower end of the interatrial septum, such as biventricular pacemaker insertion, radiofrequency ablation, are aware of these important interconnecting anastomotic vessels, to prevent a potential risk of damage.
